# Providing collaborative clinician and carer training experiences in eating disorders

**DOI:** 10.1186/2050-2974-3-S1-O30

**Published:** 2015-11-23

**Authors:** Rachel King, Belinda Caldwell

**Affiliations:** 1 Victorian Centre of Excellence in Eating Disorders, Australia

## 

The Victorian Centre of Excellence in Eating Disorders is a state-wide service providing leadership in eating disorders across the Victorian public mental health system. The service has recently employed a carer consultant to enhance their role in consultation, service development and education. One innovative use of this position was to begin delivering joint education seminars.

Five carers (who have a child with an eating disorder) were invited to participate in a focus group, informing the development of possible seminar topics. Subsequently, a senior clinician and carer consultant collaboratively designed and facilitated a pioneering seminar entitled ‘Developing a Roadmap for Recovery in Youth Eating Disorders’ to an audience of carers and clinicians. Written evaluations were completed by participants, which revealed very high satisfaction with the sessions and indicated the value of this training for both clinicians and carers.

Delivering state-wide co-produced seminars to carers and clinicians provide a valuable opportunity to encourage collaboration and empower both parties' to facilitate reciprocal sharing of expertise for continued improvement of service delivery. Further opportunities to work in partnership with carers and consumers should be encouraged, with consideration given to the benefits and challenges of developing and delivering workshops for two different audiences.

